# The Effect of Delayed Closed Reduction of Supracondylar Fracture on Perioperative Complications

**DOI:** 10.7759/cureus.32782

**Published:** 2022-12-21

**Authors:** Fahad Alshayhan, Yazeed Alsehibani, Abdulmonem Alsiddiky

**Affiliations:** 1 Orthopedic Surgery, King Saud University, Riyadh, SAU; 2 Pediatric Orthopedics, Research Chair of Spinal Deformities, Department of Orthopedics, College of Medicine, King Saud University, Riyadh, SAU

**Keywords:** trauma, gartland, complication of treatment, closed reduction, supracondylar fracture

## Abstract

Background

Supracondylar fracture is one of the most frequent pediatric traumas and surgically managed fractures. Multiple factors can contribute to delaying surgical management of supracondylar fracture, which is thought to lead to difficult reduction and more complications. Surgical treatment during the nighttime shift (from 20:00-8:00) might increase the complication rate including vascular injury, nerve injury, and the need to convert closed reduction to open due to multiple reasons including nontrained staff, exhausted on-call team, and other reasons.

Objectives

We are looking into the effect of delaying surgical intervention 24 hours from the trauma to the surgical intervention and the impact of daytime or night-time surgeries on perioperative complications.

Methods

A retrospective cohort study was conducted on all patients who presented with supracondylar fracture Gartland type 2 or 3 who required surgical intervention (63 patients) from 2018-2021 in an academic institute. All patients presented with unilateral injury. Patients were divided into an early surgical group where the surgery was done within the first 24 hours from the trauma and a delayed surgical group if performed after 24 hours. Additionally, patients were classified based on the time of the day surgery was performed into daytime or nighttime surgeries. The complication rate was compared between the groups.

Results

Most of the patients were male, and the mean age was 4.52 ± 2.28 years. No significant difference was found between the early and delayed groups in the complication rate. Nerve and vascular injury were statistically higher for cases operated at nighttime.

Conclusion

Delayed surgical treatment of supracondylar fracture doesn't affect the complication rate, whereas closed reduction of supracondylar fractures that were performed during nighttime duty was shown to lead to a higher rate of vascular and nerve injuries.

## Introduction

Supracondylar fractures are considered the most common elbow trauma in the pediatric population and represent 70% of all elbow traumas presented to the emergency department [1,2]. Children between the ages of five to eight years reported the highest incidence of supracondylar fracture and males were higher than females [2]. The typical mechanism of trauma is falling from a height on an outstretched hand but can present with direct trauma to the elbow [2]. Extension-type supracondylar fracture represents 97%, and flexion type is rare and can present as a result of a fall on a flexed elbow [3].

Treatment depends on the degree of displacement as described in Gartland’s classification: type 1 minimal displacement, type 2 intact posterior hinge, and type 3 displaced [4]. Immobilization alone for type 1 and surgery for all displaced supracondylar fractures is the standard of treatment, whereas management of type 2 is controversial [2]. The surgical intervention is a closed reduction with percutaneous wire fixation, whilst open reduction is reserved for difficult closed reduction cases [2].

Displaced supracondylar fracture carries different complications due to the surrounding neurovascular structures and proximity of fracture to the distal humerus growth plate [3,5]. Postoperative complications are either early such as neurovascular injury, compartment syndrome, or delayed such as infection, or limitation in range of motion [6,7]. 

Emergent closed reduction is necessitated in the setting of a poorly perfused limb [2]. In the presence of a well-perfused hand including a present radial pulse or warm pink hand, the safety of delayed closed reduction is still controversial [2]. Multiple factors can contribute to delay surgical intervention of supracondylar fracture including lack of instruments, availability of surgeon or anesthetist, and operating room capacity [8,9]. Delaying the management can last a couple of days depending on the institute.

We are looking in this study if delayed surgical treatment of supracondylar fracture Gartland types 2 and 3 can increase perioperative complications.

## Materials and methods

A retrospective cohort study conducted in a tertiary center from 2018-2021 included all admitted and operated supracondylar patients Gartland grade 2 and 3 [4]. The study included a total of 63 patients. Patients who presented with nondisplaced supracondylar fracture, open fracture, poor perfusion of the limb upon presentation, ipsilateral upper extremity fracture, patients diagnosed with metabolic bone disease, age above eight years, or who lost follow-up before reaching bone union were excluded. A minimum of six weeks of follow-up was considered a requirement for inclusion.

Demographic and clinical data were obtained from the patient’s electronic chart including age, gender, side of injury, time since trauma, duration from the trauma to the surgery, type of surgery, presence of nerve injury, pin site infection, compartment syndrome, need for revision, duration of follow up and final elbow range of motion. Full range of motion is considered if achieving the following criteria: flexion 130° and extension 30°. 

Radiographic data were obtained from preoperative and postoperative plain radiographs. Gartland type, fracture union, and Baumann angle were collected. Baumann angle is used to quantify the carrying angle which is made between the long axis of the humerus shaft and capitellum growth plate. The final functional outcome is measured utilizing Flynn criteria which classify the outcome from good to poor outcome based on loss of carrying angle (Baumann angle) and loss of range of motion. 

All patients were prepared and booked for surgery from their presence in the Emergency Department. Arm immobilized in the same position upon presentation with flexion < 45 degrees. Patients are divided into two groups based on the time window from the trauma to surgery: Group 1 if operated within 12 hours from trauma, and Group 2 if operated on after 12 hours from trauma. Patients under general anesthesia underwent a closed reduction in a supine position. The traction was performed initially by inline traction and counter traction. After obtaining a sufficient reduction in the fluoroscopy, the elbow is positioned in maximum flexion and pronation to lock the reduction. Fracture is either fixed with two lateral K-wires, three lateral or two lateral and one medial based on Gartland grade, presence of comminution, and stability of reduction after lateral pins placements.

The patient was immobilized on a splint and kept for 24 hours of observation for compartment syndrome, neurovascular status, and postoperative X-rays. Patients were assessed at three weeks from surgery in the clinic with X-rays to assess the union, followed by removal of K-wires at the bedside. The family was encouraged to start a gentle range of motion. At a six-week follow-up, patients were reassessed for any restriction in range motion. Then patients had a follow-up every six months to assess for any coronal deformity. Six weeks of follow-up were considered the minimum for consideration in the study.

The study was approved by the Institutional Review Board (IRB) of King Saud University, Riyadh, Saudi Arabia. Informed consent was obtained from the legal guardian of each patient. The confidentiality of each patient was maintained by converting the Medical Record Number (MRN) to coded numbers with no names. Data were analyzed using Statistical Package for the Social Sciences (SPSS) 24.0 version statistical software (IBM Corp., Armonk, NY, USA). Descriptive statistics (mean, standard deviation, frequencies, and percentages) were used to describe the quantitative and categorical variables. Bivariate statistical analysis was carried out using appropriate (chi-square, student’s t-test, one-way analysis of variance, and Pearson’s correlation) statistical tests, based on the type of study and outcome variables. A P-value of less than or equal to 0.05 and 95% CI was used to report the statistical significance and precision of the results.

## Results

The study included a total of 63 patients from 63 affected limbs. No bilateral involvement was documented. Seven patients were excluded for insufficient documentation or follow-up duration. The remaining patients were a total of 56 patients. Most of the patients were male (29; 51.8%), whereas the female group was 27 (48.2%). The mean age of the study population is 4.52 ± 2.28 years. The predominance of left-side involvement was noticed (57.1%). Based on the Gartland classification, 25 patients (44.6%) were classified as Gartland type 2, whereas 31 patients (55.4%) were Gartland type 3. The demographic data for the early intervention group and late intervention group is demonstrated in Table 1.

**Table 1:** Demographic data between the early intervention group before 24 hours from trauma and the late intervention group more than 24 hours from trauma.

	Early treatment (N=38)	Late treatment (N=18)
Average age (years)	4.16	5.28
Male	21 (72.4%)	8 (27.6%)
Female	17 (63%)	10 (37%)
Gartland type 2	14 (56%)	11 (44%)
Gartland type 3	24 (77.4%)	7 (22.6%)
Average time from presentation (hours)	3.18	28.56
Crossed pins	28	14
Two lateral pins	3	4
Three lateral pins	7	0

The average time from trauma to presentation to the emergency department was found to be 11.34 hours. The early intervention group before 24 hours from the trauma to the surgery were the majority at 38 patients (67.9%), while the delayed intervention group >24 hours from trauma to surgery were 18 patients (32.1%). The complication rate of both groups is described in Table 2. It was found that most of the patients (44 patients) were operated on during the daytime (from 08:00 to 20:00), whereas only 12 patients were operated on during the nighttime (20:00-8:00). Complication rate between daytime and night-time surgeries is described in Table 3.

**Table 2:** Describing the differences in complication rate between early intervention group before 24 hours from trauma and late intervention group more than 24 hours from trauma.

	Early treatment (N=38)	Late treatment (N=18)	P value
Open reduction	6 (15.8%)	2 (11.1%)	0.640
Pin site infection	0 (0%)	0 (0%)	
Nerve injury	3 (7.9%)	0 (0%)	0.220
Compartment syndrome	0 (0%)	0 (0%)	
Vascular injury	1 (2.6%)	0 (0%)	0.487
Revision	0 (0%)	1 (5.6%)	0.143

**Table 3:** Describing the difference in complication rate between daytime surgeries (08:00 – 20:00) and nighttime surgeries (20:00 – 08:00).

	Daytime surgery (N=44)	Nighttime surgery (N=12)	P value
Open reduction	5 (11.4%)	3 (25%)	0.231
Nerve injury	1 (2.3%)	2 (16.7%)	0.05
Vascular injury	0 (0%)	1 (8.3%)	0.05
Revision	1 (2.3%)	0 (0%)	0.598
Compartment syndrome	0 (0 %)	0 (0%)	
Pin site infection	0 (0%)	0 (0%)	

Regarding pins configuration, crossed pin construct was utilized in 42 patients (75%), three lateral pins in seven patients (12.5%), and two lateral pins in seven patients (12.5%). The selection of pins configuration was based on the presence of medial comminution and stability of the fracture. No statistical significance was found between the type of construct and the rate of complications including nerve injury, vascular injury, and pin tract infection. Certainly, all three nerve injury patients were ulnar nerve and found in crossed pins construct group without statistical significance.

Failed closed reduction was observed in eight patients, two of them (2%) were classified to have Gartland type 2 and six of them (19.4%) were classified as Gartland type 3 without statistical significance. All of them underwent revision closed reduction and percutaneous pinning. Only one patient where the open reduction was utilized with co-existent vascular injury, whereas others have no vascular injury.

All our patients presented with palpable pulses and well-perfused hands. In one patient during reduction, the pulse wasn’t palpable, and the hand became cold when open vascular exploration was necessitated and performed. Nerve injury happened in three patients (5.4%); all were among the early intervention group and all injuries were ulnar nerve palsy which resolved spontaneously. No pin site infection or compartment syndrome was reported among patients. Surgery was revised with a closed reduction in one patient from the early intervention group due to loss of reduction from post-operative radiographs. The patient presented with Gartland type 2 supracondylar fracture and the initial procedure was performed during the daytime.

The final elbow range of motion was limited in four patients (7.1%). Three patients were Gartland type 3 and one patient was Gartland type 2 without statistical significance. Two patients (25%) required open reduction and ended up with a restricted range of motion with statistical significance (0.035). The final functional outcome was assessed by using Flynn’s criteria where a satisfactory excellent outcome is considered when the loss of carrying angle is between 1-5 degrees and/or 0-5 degrees in motion loss. Satisfactory good outcome scored if loss of carrying angle is between 6-10 degrees and/or 6-10 degrees in motion loss. A loss of carrying angle between 11-15 degrees and/or 11-15 degrees in motion loss is considered a satisfactory fair outcome. Lastly, The unsatisfactory outcome is considered when the loss of carrying angle is more than 15 degrees and/or motion loss is more than 15 degrees.

Regarding Flynn’s criteria, 50 patients (89.3%) showed satisfactory excellent outcomes, one patient (1.8%) was satisfactory good, two patients (3.6%) were satisfactory fair, and three patients (5.4%) showed unsatisfactory outcomes. No clinical significance was found between Flynn’s groups and the timing of surgical intervention. Clinical outcome difference between the early and late surgical group in form of Flynn's criteria is described in Table 4.

**Table 4:** Describing Flynn’s classification and the difference between early intervention group before 24 hours from trauma and late intervention group more than 24 hours from trauma.

	Early intervention group	Late intervention group
Satisfactory Excellent N (%)	34 (68%)	16 (32%)
Satisfactory Good N (%)	1 (100%)	0 (0.0%)
Satisfactory Fair N (%)	1 (50%)	1 (50%)
Unsatisfactory N (%)	2 (67.9%)	1 (32.1%)

## Discussion

Supracondylar fractures are one of the most prevalent fractures in the pediatric age group. Our aim in this research is to study the effect of delaying surgical management on perioperative complications. Our sample size is 56 patients, results were categorized into the early intervention group (38 patients) and the late intervention group (18 patients). Failure of closed reduction and need for open reduction was observed in eight patients in total. 15.8% of our patients that underwent early intervention required open reduction and 11.1% of the patients that underwent late intervention group experienced this outcome as well. However, this difference was not statistically significantly similar to what other published articles in the literature have stated [2,5,6,10-15]. This may indicate that delayed surgical management does not increase the incidence of open reduction, unlike another systematic review which stated that the need for open reduction was significantly lower in the early management group [14] as well as another study that showed an increased rate of open reduction if the management was delayed in case of Gartland type 3 fractures [16]. We also observed that the majority of patients who underwent open reduction were Gartland type 3 fractures which also may explain the tendency for open reduction rather than the timing of surgery. Out of 56 cases, the vascular injury was documented in one patient in the early intervention group, which is not statistically significant, this could be due to the fact the fracture was Gartland type 3 since displaced fractures carry a higher risk of neurovascular complications [3,6]. On the other hand, the fact that there is only one recorded case of vascular injury could be attributed to the level of care and experience in our institution.

The timing of surgery and the difference between daytime (08:00 - 20:00) and nighttime (20:00 - 08:00) in terms of complications were also studied. Neurovascular injuries are more prevalent after nighttime surgeries with statistical significance. Similarly, another study showed a higher incidence of paresthesia after nighttime surgeries [5]. Generally, a higher incidence of surgical errors and postoperative complications was observed when the surgery was done at night compared to daytime [2,5,17,18] and these results can be explained by many factors such as experienced staff availability or team focus and fatigue for example.

Nerve injury is one of the reported complications of supracondylar fracture in the pediatric age group. In our study, nerve injury occurred in 7.9% (three patients) of cases in which all are from the early intervention group and the nerve injury diagnosed was ulnar nerve palsy after closed reduction. All of the patients had neuropraxia injuries and experienced spontaneous resolution with follow-up. These results are consistent with other studies [3,6,8] indicating that delayed surgical management does not increase the risk of nerve injury. Moreover, in all three patients who had ulnar nerve palsy, a crossed-pin configuration was used during closed reduction similar to previous studies [3,10] where the incidence of ulnar nerve palsy was higher in crossed-pinning compared to lateral pins configuration, unlike another study in which showed no correlation between the two [17]. Our results could be explained by the vulnerability and proximity of the ulnar nerve and medial pin which may increase the risk of injury during placement.

Pin site infection and compartment syndrome were also known complications of interest in our study. Compared to other studies in the literature [3,5,6,8,11], we didn’t have any cases of pin site infection or compartment syndrome, thus we could not study the effect of delaying surgical management on the incidence of either of them. Yet, a multicenter study showed that compartment syndrome was seen in 11 patients who had low energy, closed supracondylar fracture and the authors assumed that delayed surgical management contributed to the development of compartment syndrome [19]. On the other hand, in all previous three studies [3,6,8], the infection rate was not statistically significant between both early and late intervention groups to favor early surgical management. In contrast, the absence of pin site infection cases in our study can be explained by the relatively smaller sample size compared to other studies or also can be rewarded by the septic techniques which have been followed in our center.

The need for revision after initial surgical management was seen in one case only in the late intervention group which is not statistically significant as seen in another study [3] that showed no difference between early and late treatment groups in this regard. This also could validate the conclusion that delaying surgical management does not increase the risk of revision after initial surgical management.

The final elbow range of motion was one of the secondary objectives of our study. We found out that it was restricted to a total of six patients of which two of them required open reduction surgery with statistical significance. Furthermore, we consider the limitation in range of motion of elbow joint post open reduction surgery is at a higher risk compared to the closed reduction contrary to another article which states the opposite [2] as well as another study which showed that the outcome in both closed and open reduction was similar [15]. In contrast, delayed surgical management does not increase the risk of open-reduction surgery based on our results. Flynn’s criteria were used as a measuring tool to assess the final functional outcomes after surgical management which showed no statistical significance between early and late intervention groups on the outcomes, this is in line with other studies which concluded that there is no correlation between delaying surgical management and unsatisfactory outcome [6,12,13,20-23].

## Conclusions

In conclusion, early surgical treatment for supracondylar fractures did not have an impact on perioperative complications compared to delayed management based on our results. Nighttime surgeries carry a higher risk for complications; therefore, we recommend performing closed reduction during daytime in the presence of well-perfused limbs. The need for open reduction might affect the final range of motion elbow.
